# Evaluation of HER2/neu expression in different types of salivary gland tumors: a systematic review and meta-analysis

**DOI:** 10.25122/jml-2021-0394

**Published:** 2022-05

**Authors:** Aylar Javaheripour, Maedeh Vakili Saatloo, Nafiseh Vahed, Leili Faraji Gavgani, Maryam Kouhsoltani

**Affiliations:** 1. Department of Oral and Maxillofacial Pathology, Faculty of Dentistry, Tabriz University of Medical Sciences, Tabriz, Iran; 2. Department of Microbiology and Immunology, Medical University of South Carolina, Charleston, South Carolina, United States of America; 3. Research Center for Evidence Based Medicine, Tabriz University of Medical Sciences, Tabriz, Iran

**Keywords:** salivary gland, cancer, HER2, oral pathology, carcinoma, ACC – Adenoid Cystic Carcinomas, HER2 – Human epidermal growth factor receptor 2, MEC – Mucoepidermoid Carcinoma, PRISMA – Preferred Reporting Items for Systematic Review and Meta-analyses

## Abstract

This study is a systematic review and meta-analysis to assess the overexpression rate of HER2 in patients with salivary gland tumors. We included peer-reviewed publications from 1995 to 2020, indexed in medical databases, using search terms such as “human epidermal growth factor receptor 2 (HER2)” and “salivary gland tumors”, and extracted relevant data. The extracted data were analyzed with RevMan 5.3 software. Intra-and intergroup post hoc analyses of outcome variables were performed using t-tests, and the rates of HER2 positivity among studies were evaluated. 80 studies were included in the analysis. The positive rates of HER2 ranged from 3.3% to 84.0% and 1% to 9% in malignant and benign subtypes, respectively. The highest HER2 overexpression rate among malignant tumors was in salivary ductal carcinomas (SDC), with a 45% positive rate (CI 95%: 21.9–70.3%). Mucoepidermoid carcinoma (MEC) had the highest positive rate of 84% (CI 95%: 74.1–90.0%). Among benign salivary gland tumors, the highest rate was found in myoepithelioma, with a positive rate of 9% (CI 95%: 1.7–33.6%). The highest rate of HER2 overexpression is present in malignant subtypes of salivary gland tumors, more specifically in salivary ductal carcinoma, mucoepidermoid carcinomas, salivary duct carcinoma in situ, and carcinoma ex pleomorphic adenoma.

## INTRODUCTION

Benign neoplasms comprise the major part of salivary gland tumors. However, salivary gland malignancies make up about 11% of all head and neck cancers, with more than 20 pathological subtypes [1–3]. This heterogeneity challenges the treatment of these carcinomas [4], and although adjuvant therapy following surgery is the treatment of choice in most cases, the results are usually not favorable [5, 6]. Nevertheless, since the emergence of modern, effective molecular targeted therapies for various types of cancers [7], such as breast cancer, non-small cell lung cancer, acute myeloid leukemia, renal cell carcinoma, and hepatocellular carcinoma [8–12] and successful application of trastuzumab and pertuzumab, which target human epidermal growth factor Receptor 2 (HER2 also known as HER2/neu), in management of the patients with HER2-positive metastatic breast cancer [8], numerous studies tried to demonstrate the efficacy of these group of medications in the treatment of other HER2/neu positive cancers. HER2/neu is a proto-oncogene that encodes a transmembrane glycoprotein – with a tyrosine kinase activity and similarities to epidermal growth factor receptor [13–15]. In addition to breast cancer, its overexpression/amplification has been detected in various malignant neoplasms such as ovarian, gastrointestinal, and salivary gland cancers [16–19]. Several studies have immunohistochemically assessed salivary gland tumors for overexpression of HER2/neu; however, the results are somewhat controversial [20–24]. For instance, the HER2 overexpression rate was reported to vary between 38% to 67% in mucoepidermoid carcinomas (MEC) and 30% to 77% in adenoid cystic carcinomas (ACC) in different studies. Also, there is a lack of data reporting the pooled results of HER2 overexpression rates in salivary gland tumors, leading to a new paradigm for future studies to evaluate possible therapeutic options for this family of neoplasms. The present study is a systematic review and meta-analysis of the expression of HER2/neu in different types of salivary gland tumors.

## MATERIAL AND METHODS

This systematic review was performed based on the PRISMA guidelines for writing systematic reviews.

### Search strategy and study selection criteria

We performed an electronic search on Pubmed (Medline), Scopus, Proquest, The Cochrane Library, Psycoinfo, and CINAHL databases, including studies from 1995 to 2020. The search strategy was based on Medical Subject Headings (MeSH) and a combination of free words in the title/abstract as follows: (human epidermal growth factor receptor (HER/EGFR/ERBB) “ErbB2”, “c-erb2”, “EGFR2”, “erbB-2 proto-oncogene protein”, “tyrosine-protein kinase erbB2 receptor”, and “human epidermal receptor 2”. A manual search of articles presented in related conferences was performed. The search was restricted to English-language articles. Studies were eligible for inclusion if HER2 expression was detected by immunohistochemistry (IHC) with or without fluorescence in situ hybridization. The following articles were excluded: (1) not peer-reviewed; (2) not published in full (3) review articles, letters to editor, commentaries, case reports; (4) non-English language articles; (5) articles from which the relevant data could not be extracted; (6) non-human research (7) articles of poor quality; (8) studies without qualitative staining and immunohistochemistry analysis; and (9) duplicated articles (risk of bias). Articles reporting less than three cases were not included.

### Data Extraction

One of the authors selected the potentially eligible studies, and then two specialists in oral and maxillofacial pathology independently reviewed the quality of the articles using the Critical Appraisal Skills Program (CASP) quality checklist, and low-quality studies were excluded. Data were extracted by the first author (AJ). If there were duplicates, the most informative cohort was included. To ensure the review was comprehensive and reduce the risk of introducing selection bias, we aimed to include studies irrespective of the immunohistochemistry (IHC) methods used to measure overexpression of HER2/neu. The extracted data for each study included the first author's name, publication year, type of the study, number of patients, detection methods, rate of HER2 positivity, tumors' malignancy or benignity, and types of tumors.

Endnote X5 Resource Management Software was used to organize titles and abstracts of the articles and to recognize duplicates.

## ANALYSIS

The comprehensive meta-analysis (CMA; Englewood, NJ, USA), software version 2.0, was used to analyze the data. Q statistics and I^2^ were used to purpose heterogeneity. A possibility value of P-value<0.10 and I^2^ value >50% for Cochran's Q showed the existence of noteworthy heterogeneity. According to the analysis, fixed or random-effects models were used. We used a fixed-effect model with a 95% confidence interval and significance level of 0.05 to perform the meta-analysis and forest plots to report the results. To check for publication bias, funnel plots and Egger's test with a significance level of 0.01 were used.

## RESULTS

### Study selection and characteristics

**Figure 1:**
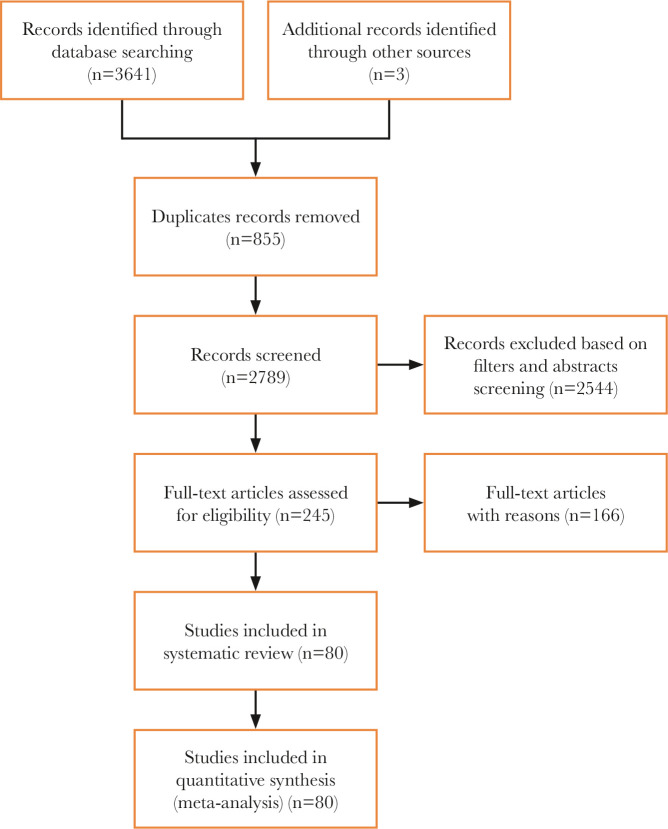
PRISMA flow diagram.

The online database search identified 3641 publications, and 3 more were identified from article lists. 855 publications were duplicates. All abstracts were reviewed, and 245 papers were read in full. The list of articles is available on request from the authors. 80 papers fulfilled the criteria for the study and were included in the analysis. The flow diagram shows the article selection process (Figure 1).

Studies were from South Korea (7), Germany (8), Japan (13), the United States (15), Canada (3), Brazil (5), Iran (1), the United Kingdom (2), Italy (5), Israel (2), Poland (2), Taiwan (1), Czech Republic (2), France (2), the Netherlands (2), China (2), Australia (1), Greece (2), Finland (3), and Portugal(2).

37 of the included studies were retrospective cohort studies, 33 were cross-sectional studies, 4 were clinical trials, 3 were case series, and 3 were case-control studies. Based on the quality criteria, all the studies were of high quality.

### Study results and meta-analysis

Of 80 studies, 8 reported the expression of HER2/neu in benign tumors, 78 studies reported the expression of HER2/neu in malignant tumors, 65 used evaluation methods for IHC used to assess HER2 in malignant salivary gland tumors (Group 1), and the remaining 13 used a variety of methods with different cut off points and scoring scales (Group 2).

### Malignant salivary gland tumors (Group 1)

Of 65 studies, overexpression of HER2/neu in salivary ductal carcinomas (SDC) was assessed in 40 studies, making it the most studied salivary gland tumor in this meta-analysis. The least studied salivary gland tumors were cystadenocarcinoma and poorly differentiated adenocarcinoma, with 1 study pertaining to each of them. After pooling the data for each salivary gland tumor subtype, the highest heterogeneity was seen in studies related to adenoid cystic carcinomas (ACC) (I^2^=86.126, P=0.002) and then in the studies related to SDC (I^2^=77.950, P=0.000). More details are available in Table 1.

**Table 1:** Number of studies assessing the expression of HER2/neu protein in salivary gland tumors, the rate (%) and 95% confidence interval of expression, and the level of heterogeneity in the studies related to each subtype.

Tumor subtype	Number of studies	Expression of HER2/neu protein (%)	95% CI		I-squared	P-value
			Lower limit (%)	Upper limit (%)		
**Acinic cell carcinoma**	12	16.2	10.6	24.0	62.957	0.002
**Adenocarcinomas, not otherwise specified (NOS)**	9	22.3	15.5	31.0	0.000	0.667
**Adenoid cystic carcinoma (ACC)**	14	17.2	12.1	24.0	86.126	0.000
**Basal cell adenocarcinoma (BCAC)**	4	4.7	1.2	17.3	0.000	0.602
**Salivary duct carcinoma in situ**	2	44.9	21.9	70.3	53.673	0.142
**Cystadenocarcinoma**	1	3.3	0.2	36.6	0.000	1.000
**Epithelial-myoepithelial carcinoma (EMC)**	4	5.2	1.5	16.5	0.000	0.596
**Carcinoma ex pleomorphic adenoma (CXPA)**	14	33.7	28.8	38.9	46.857	0.027
**Polymorphous low-grade adenocarcinoma (PLGA)**	3	4.0	0.8	17.9	0.000	0.529
**Mucoepidermoid carcinoma (MEC)**	15	12.9	10.2	16.1	54.712	0.006
**Myoepithelial carcinoma**	4	5.1	1.5	16.2	0.000	0.908
**Oncocytic carcinoma**	2	4.6	0.6	26.5	0.000	0.851
**Poorly differentiated adenocarcinoma**	1	25.0	3.4	76.2	0.000	1.000
**Salivary ductal carcinoma (SDC)**	40	38.1	35.3	41.1	77.950	0.000
**Squamous cell carcinoma**	4	21.4	8.2	45.4	48.320	0.121

The meta-analysis showed that salivary duct carcinoma in situ had the highest pooled overexpression rate of HER2/neu among salivary gland tumors, 45% (CI 95%: 21.9–70.3%). Also, the lowest expression rate was for cystadenocarcinoma, with an expression rate of 3.3% (CI 95%: 0.2–36.6%).

### Malignant Salivary gland tumors (Group 2)

In the studies evaluating IHC with a cut-off point of >30% for +3 positive, >10% for +2, +1 for faint and barely, and 0 for negative results, tumors with ≥10% nuclear-stained cells were considered positive. The positive rate in salivary duct carcinoma in situ was 23% (CI 95%: 11.9–39.5%), the expression rate of HER2/neu in carcinoma ex pleomorphic adenoma (CXPA) was 55% (CI 95%: 37.4–71.1%), and the pooled positive rate of for SDC was 41% (CI 95%: 34.8–47.7%).

In the studies where the cut-off point was >50% for strongly positive, 5–50% weak positivity, and <5% for negative results, the expression rate of HER2/neu in SDC was 30% (CI 95%: 15.6–49.0%). Also, the summary expression rate of HER2/neu in MEC was 84% (CI 95%: 74.1–90.0%).

Finally, in the studies with a cut-off point of 50% for positive and 10% for negative results, the overexpression rate of HER2/neu in SDC was 33% (CI 95%: 13.1–62.4%), and the expression rate of HER2/neu in ACC was 2% (CI 95%: 0.2–31.0%).

### Benign salivary gland tumors

In benign tumors of the salivary gland, basal cell adenoma had an overexpression rate of HER2/neu of 2% (CI 95%: 0.1–27.7%), warthin tumor had an expression rate of 1% (CI 95%: 0.1–13.1). For myoepithelioma the summary expression rate of HER2/neu was 9% (CI 95%: 1.7–33.6%). Ultimately, the summary expression rate of HER2/neu for pleomorphic adenoma was 5% (CI 95%: 1.9–10.7%).

### Publication Bias

For publication bias analysis, a funnel plot and Egger's test with a P-value<0.1 were used. In malignant salivary gland tumors, studies involving SDC (p=0.001), squamous cell carcinoma (p=0.059), an acinic cell carcinoma (p=0.015), basal cell adenocarcinoma (BCAC) (p<0.001) and ACC (p=0.005) had significant publication biases.

There was no statistically significant publication bias for SDC in studies assessing the overexpression rate of HER2/neu using a cut-off point of 30% and 10% (p=0.98).

Among the studies assessing the expression rate of HER2/neu using a cut-off point of 10% and 50%, studies related to MEC had no statistically significant publication bias (p=0.90).

For benign salivary gland tumors, results reporting the rate of HER2/neu overexpression in myoepithelioma had no statistically significant publication bias (p=0.060), and studies assessing the expression rate of HER2/neu in pleomorphic adenomas had a statistically significant publication bias (p=0.06).

## DISCUSSION

HER2 overexpression has been studied as a prognostic factor as well as a target for therapy in some malignancies such as breast cancer [23]. Several studies found that despite the worse prognosis of HER2 overexpressing tumors, targeted treatment with monoclonal antibodies such as Lapatinib and Trastuzumab resulted in prolonged event-free periods and overall survival rates. Nevertheless, several studies tried to evaluate the impact of HER2/neu overexpression on the prognosis of salivary gland tumors and showed that overexpression of HRE2/neu is a marker of poor prognosis independent of histopathological grade and tumor size [24–26].

In the present systematic review and meta-analysis, we found that 6 subtypes of malignant salivary gland tumors had positive HER2 rates higher than 20%, including salivary duct carcinoma in situ, SDC, CXPA, poorly differentiated adenocarcinoma, adenocarcinomas not otherwise specified (NOS), squamous cell carcinomas and mucoepidermoid carcinomas. Furthermore, HER2/neu overexpression was more prevalent in malignant salivary gland tumors than benign tumors. The rates varied among different subtypes of malignant tumors with different histopathological characteristics and levels of invasiveness, with SGCs having a high expression of HER2, specifically subtypes other than SDC and CXPA.

Similarly, Giannoni et al. [27], in a study of minor salivary gland tumors of the palate, reported that gender, tumor stage, and HER2/neu overexpression together are predictors of survival in patients with malignant tumors. The highest rate of overexpression of HER2/neu in benign tumors in this study was 9% for myoepithelioma, in contrast to malignant tumors, which had rates as high as 84% in MECs. Pooled positive rates of HER2/neu overexpression in CXPA tested by different IHC methods were 55% and 33.7%. For SDCs, the positive overexpression rate ranged from 30% to 41% based on the IHC method used.

Similar to the findings of our analysis, studies showed a significant difference in overexpression of HER2/neu between benign and malignant tumors [27]. Even though not all malignant salivary gland tumors showed high HER2/neu overexpression, the difference between overexpression in benign and malignant tumors can suggest an association between overexpression of HER2/neu and malignancy of the tumor.

There is an association between overexpression of HER2/neu and aggressive behavior in tumors [27]. This may explain the higher rate of overexpression in SDCs than other less aggressive subtypes of salivary gland tumors such as ACCs. Also, Shintani et al. [21], in a study of patients with ACCs using IHC, observed strong staining mostly in cells of the invasive area. That being said, metastatic potential and tumor recurrence were also associated with HER2/neu overexpression [28]. Differences in the overexpression rates of HRE2/neu were also observed in tumors of the same subtype but in different areas of origin [29–32].

Similar to breast cancer, studies have shown promising results in treating salivary gland cancers with a high HER2/neu overexpression, such as SDC and CXPA, with targeted therapy having HER2/neu as a molecular target [33–36]. For example, a recent phase II clinical trial assessing the efficacy and toxicity of trastuzumab plus docetaxel in 57 patients with locally advanced and/or recurrent or metastatic HER2+ SDC yielded a 70.2% overall response rate with a clinical benefit rate of 84.2%. This led Takahashi et al. [37] to conclude that this regimen is effective in treating HER2+ SDCs. Although there are studies with contrasting results, multiple factors, such as a small number of included patients in the studies, make it difficult to draw a conclusion [38–42].

These results suggest the possibility of promising outcomes for patients with HER2+ salivary gland tumors being treated with targeted therapy. In addition to this, we can conclude that anti-HER2 therapy may be effective for treating patients with other subtypes of salivary gland tumors with a high overexpression of HER2/neu. However, a series of carefully designed studies are required to reach a consensus.

Notably, HER2/neu positivity ranges vary between studies, which can be due to several factors. Besides the tumor heterogeneity, the impreciseness of techniques used to detect HER2/neu overexpression can be a cause for this variability. This inconsistency in HER2 testing results is due to various reasons, including limitations in IHC techniques and a lack of uniform cut-off values and protocols. While there is a HER2 testing guideline for breast cancers [43], there are no definitive algorithms for defining positive, negative, and equivocal values for HER2/neu expression in salivary gland tumors. There are multiple cut-off points for IHC overexpression which vary from 10% [44] and 30% [45] to 50% [46]. A universal guideline suggesting a uniform approach to IHC in salivary ductal tumors can resolve this issue.

There are a number of limitations in this meta-analysis. The first limitation is publication bias. We used funnel plot determination to evaluate publication bias and performed Egger's test to confirm the results. However, each study included in this meta-analysis is susceptible to bias. For instance, we only included the published English articles. Positive studies are accepted more commonly in English language journals, yet studies yielding negative results, more often than not, are submitted to native journals. Also, including only full published studies and excluding studies with incomplete data may cause bias.

Heterogeneity is the second limitation, as high heterogeneity was observed among the studies in regards to the rate of HER2/neu overexpression. Several factors can result in heterogeneity among studies. For example, different baseline characteristics in patients (such as age, ethnicity, tumor stages), differences in methods of sampling, and their effects on IHC detection, such as fragmentation during the biopsy process, can increase the edge effect, thus causing stronger staining at the circumference, differences in materials used in the process.

Also, because only a limited number of studies are eligible, some of the studies had relatively small sample sizes and a weaker level of evidence.

Finally, the complexities in the mechanisms involved in tumorigenesis and the relationship between various factors determining the expression of certain proteins and tumor invasiveness should be considered. Consequently, the effectiveness of HER2-targeted therapy must be further examined. The results of this study showed that HER2/neu overexpression was associated with a high level of invasiveness and malignancy of the tumor. Therefore, functions of HER2 in the aggressive behavior of certain types of salivary duct carcinomas must be further investigated.

## CONCLUSION

To our knowledge, this study is the first systematic review and meta-analysis that evaluated the overexpression rates of HER2/neu in patients with various subtypes of salivary gland tumors. The results showed that HER2/neu overexpression was more prevalent in malignant salivary gland tumors than benign tumors. Also, the rates varied among different subtypes of malignant tumors with different histopathological characteristics and levels of invasiveness. Also, the results suggest the possibility of using novel targeted therapy regimens for treating patients with SGCs having a high expression of HER2, specifically subtypes other than SDC and CXPA. Having said that, the results of this systematic review should be carefully interpreted regarding the discussed limitations. Therefore, large-high-quality prospective studies must be performed to confirm the results.
